# Vangl2 regulates spermatid planar cell polarity through microtubule (MT)-based cytoskeleton in the rat testis

**DOI:** 10.1038/s41419-018-0339-x

**Published:** 2018-03-01

**Authors:** Haiqi Chen, Xiang Xiao, Wing-yee Lui, Will M. Lee, C. Yan Cheng

**Affiliations:** 1 The Mary M. Wohlford Laboratory for Male Contraceptive Research, Center for Biomedical Research, Population Council, 1230 York Ave, New York, NY 10065 USA; 2 Department of Reproductive Physiology, Zhejiang Academy of Medical Sciences, Hangzhou, Zhejiang 310013 China; 3 School of Biological Sciences, University of Hong Kong, Hong Kong, China

## Abstract

During spermatogenesis, developing elongating/elongated spermatids are highly polarized cells, displaying unique apico-basal polarity. For instance, the heads of spermatids align perpendicular to the basement membrane with their tails pointing to the tubule lumen. Thus, the maximal number of spermatids are packed within the limited space of the seminiferous epithelium to support spermatogenesis.  Herein, we reported findings that  elongating/elongated spermatids displayed planar cell polarity (PCP) in adult rat testes in which the proximal end of polarized spermatid heads were aligned uniformly across the plane of the seminiferous epithelium based on studies  using confocal microscopy and 3-dimensional (D) reconstruction of the seminiferous tubules.  We also discovered  that spermatid PCP was regulated by PCP protein Vangl2 (Van Gogh-like protein 2) since Vangl2 knockdown by RNAi was found to perturb spermatid PCP. More important, Vangl2 exerted its regulatory effects through changes in the organization of the microtubule (MT)-based cytoskeleton in the seminiferous epithelium. These changes were mediated via the downstream signaling proteins atypical protein kinase C ξ (PKCζ) and MT-associated protein (MAP)/microtubule affinity-regulating kinase 2 (MARK2). These findings thus provide new insights regarding the biology of spermatid PCP during spermiogenesis.

## Introduction

Spermatogenesis takes place in the seminiferous tubules, the functional unit in the testis that produces ~8, 70, and 200 million of sperm daily from a normal adult male mouse, rat and human, respectively^1–3^. This thus represents an enormous cellular output wherein millions of developing spermatids are packed across the seminiferous tubules in the seminiferous epithelium to support spermatogenesis^4,5^. Thus, spermatids are orderly arranged in the limited space of the seminiferous epithelium to be supported by Sertoli cells—the only somatic cells in the seminiferous epithelium to sustain spermatogenesis. Studies in the testis have shown that spermatid heads align perpendicularly to the basement membrane of the seminiferous epithelium, with their tails pointing toward the tubule lumen^6–9^. This apico-basal polarity of spermatids is supported by the Par^10^, Scribble^11^, and CRB3-based^12^ polarity protein complexes through their effects on the testis-specific anchoring junction ectoplasmic specialization (ES), which is the only anchoring device at the Sertoli-spermatid (step 8–19) interface in the rat testis. Interestingly, when cross-sections of stage VII–VIII tubules were examined, step 19 spermatids were found to display polarity not just along the apico-basal axis of the seminiferous epithelium. Heads of elongated spermatids also exhibit a uniform polarized alignment within the plane of the seminiferous epithelium, resembling the planar cell polarity (PCP) that is remarkably noted in wing cell hair in *Drosophila*, and cell hair of the inner ear in mammals^13–15^. This thus prompted us to examine the involvement of PCP proteins in spermatid polarity.

PCP refers to the alignment of a field of polarized cells within the plane of an epithelium. PCP proteins were originally identified as initiators and regulators of PCP^16^. Recent studies, however, have shown that many PCP proteins exert other functions beyond conferring PCP. These include the involvement of PCP proteins in the development and functioning of the nervous system^17–19^, kidney^20,21^, lung^22^, skin^23^, female reproductive tract^24^, and heart^25^. They also play a crucial role in tumorigenesis^26,27^. Recently, we have reported elongating/elongated spermatids also displayed PCP in adult rat testes in which the proximal end of polarized spermatid heads were aligned uniformly across the plane of the seminiferous epithelium by confocal microscopy and 3-dimensional (D) reconstruction of the seminiferous tubules^28^. Studies also have identified several PCP genes/proteins in the mammalian testis^29–32^. Among these, Vangl2 was found to regulate the apical ES at the spermatid-Sertoli cell interface and at the Sertoli cell–cell interface at the blood-testis barrier (BTB) called basal ES^31^. The regulatory effects of Vangl2 on ES dynamics are mediated, at least in part, via changes in F-actin organization by modulating the spatio-temporal expression of actin-regulatory proteins Arp3 and Eps8. Interestingly, the knockdown of Vangl2 by RNAi in the testis in vivo induced extensive defects in spermatid polarity most notably in stage VII–VIII tubules^31^. This thus prompted us to investigate the role of Vangl2 on spermatid PCP. Studies have shown that the microtubule (MT)- and actin-based cytoskeletal networks are working in concert to support spermatid transport and polarity in the testis^33–36^. Emerging evidence illustrates that PCP proteins are involved in regulating MT dynamics (i.e., assembly, disassembly, and stabilization/maintenance). For instance, dishevelled, another PCP protein, has been shown to facilitate axon formation of hippocampal neurons by stabilizing and activating atypical protein kinase C (aPKC) PKCζ, which in turn inhibits MAP/microtubule-affinity regulating kinase 2 (MARK2) activity to promote MT stability^37^. Also, Vangl2 has been shown to interact with aPKC, and the expression of Vangl2 overlapped with that of stabilized MTs such as acetylated MTs in *Xenopus* oocytes to support oocyte maturation^38^. Herein, we sought to examine the role of Vangl2 in spermatid PCP, and if Vangl2 exerts its regulation through PKCζ and MARK2 downstream.

## Materials and methods

### Animals

Sprague-Dawley male pups at 20 days of age (a foster mother was shipped with 10 pups), and adult male rats of 250–300 gm b.w. were obtained from Charles River Laboratories (Kingston, New York). All rats were  housed at the Comparative Bioscience Center (CBC) of The Rockefeller University with ad libitum access to standard rat chow and water under controlled temperature (21 ± 1 °C) and constant light-dark cycles (12 h of light and 12 h of darkness). The Rockefeller University CBC animal facilities have been fully accredited by the American Assocaiton for Accreditation of Laboratory Animal Care. Rats were maintained in accordance with the applicable portions of the Animal Welfare Act and the guidelines in the Department of Health and Human Services Publication Guide for the Care and Use of Laboratory Animals. The use of rats in this report was approved by The Rockefeller University Institutional Animal Care and Use Committee with Protocol Numbers 12-506 and 15-780-H. The use of recombinant DNA (e.g., plasmid DNA) or synthetic nucleic acids (e.g., siRNA duplexes) for studies has been approved by the Rockefeller University Institutional Biosafety Committee (IBC) with Protocol Number 2015-04-007. At specified time points, rats were euthanized by CO_2_ asphyxiation using slow (20–30% per min) displacement of chamber air with compressed CO_2_.

### Primary Sertoli cell cultures

Sertoli cell cultures were prepared using cells isolated from 20-day-old rat testes as detailed elsewhere^39^. Cells were plated on Matrigel (BD Biosciences, dilution 1:7 in F12/DMEM medium)-coated dishes or cover glasses (round, 18-mm diameter) at different densities optimized for specific experiments based on pilot experiments as follows. For the preparation of cell lysates for immunoblotting and microtubule (MT) spin-down assays, Sertoli cells were plated at 0.4×10^6^ cells/cm^2^ on 6-well dishes containing 5-ml F12/DMEM. For dual-labeled immunofluorescence (IF) analysis, Sertoli cells were cultured at 0.04×10^6^ cells/cm^2^ on microscopic cover glasses, and cover glasses were placed on 12-well dishes with each well containing 2-ml F12/DMEM.

### RNA interference (RNAi)

#### Studies in vitro

Vangl2 RNAi performed in primary Sertoli cell culture was described in details elsewhere^31^. In brief, primary Sertoli cells were transfected with Silencer Select Negative Control No.1 siRNA (Ambion-Thermo Fisher Scientific) (Ctrl) vs. Silencer Select siRNA duplexes specifically targeting rat Vangl2 (s144160 and s144162, Ambion- Thermo Fisher Scientific) at 100 nM using Lipofectamine RNAiMax (Invitrogen-Thermo Fisher Scientific) as a transfection medium according to the manufacturer’s instructions on day 2 and 4, respectively, with a 12 h recovery in-between. In short, cells were transfected twice, which was based on results of pilot experiments that a double knockdown was necessary to silence the expression of Vangl2 by ~70%. Thereafter, cells were terminated on day 5. The sequences of the two pairs of Vangl2 siRNA duplexes used for our studies were: sense: 5′-GGCACUUCUGAGCACAGUAtt-3′, antisense: 5′-UACUGUGCUCAGAAGUGCCtg-3’ (s144160); and sense: 5′-AGGAAUUCGUGGAUCCCAAtt-3′, antisense: 5′-UUGGGAUCCACGAAUUCCUcg-3 (s144162). MARK2 RNAi was performed similar to Vangl2 RNAi except that specific MARK2 siRNA duplexes from the SMARTpool (Dharmacon-GE Healthcare) were used with the following sequences: 5′-GCGAGCUGCACGAGCGAUA-3′; 5′-GCACAGAGUAUUUCGCCUA-3’; 5′-GGAAGAGACAGGGCGGAAA-3′ and 5′-UCUCAACGGUGUUCGGUUU-3’. For IF cell staining, siGLO Red Transfection Indicator (Dharmacon-GE Healthcare) was co-transfected with either Ctrl, Vangl2 or MARK2 siRNA duplexes at 1 nM to illustrate successful transfection. In selected in vitro experiments, successful transfection was confirmed by transfecting siRNA duplexes labeled with Cy3 dye using Label IT® siRNA Tracker Intracellular Localization Kit (Mirus) (Figure S1). For dual silencing of Vangl2 and MARK2, both siRNA duplexes were used for simultaneous transfection (i.e., 100 nM each, to a total of 200 nM siRNA duplexes) vs. the same concentration of non-targeting negative control siRNA duplexes (i.e., 200 nM) in Sertoli cells using the treatment regimen described above.

#### Studies in vivo

Vangl2 RNAi in vivo was performed, as earlier described^31^. In brief, both non-targeting negative control and Vangl2 siRNA duplexes were labeled with Cy3 dye using Label IT® siRNA Tracker Intracellular Localization Kit (Mirus), forming the Cy3-labeled siRNA duplexes which was used to indicate successful transfection (Figure S1). In short, labeled siRNA duplexes obtained using this kit were re-suspended in 10  µl sterile water. Both non-targeting control and Vangl2 siRNA duplexes stocks were diluted in sterile 5% glucose (wt/vol) and mixed with the in vivo-jetPEI reagent (Polyplus) (i.e., at 0.16  µL in vivo-jetPEI reagent per µg siRNA duplexes) at an N/P ratio of 6 (note: N/P ratio is a measure of the ionic balance of the nucleotide, and it refers to the number of nitrogen residues of jetPEI/nucleotide phosphate, in which the jetPEI concentration is expressed in nitrogen residues molarity in which 1 µg of nucleotide contains 3 nmol of anionic phosphate) to reach a mix volume of 70 µl for each testis. It was noted that the transfection efficiency using PolyPlus in vivo-jetPEI reagent was estimated to be at least ~60% as earlier reported from our laboratory^40,41,]^, which is considerably higher than a regular transfection medium^42^. In short, each testis of the same rat received either 100 nM negative control siRNA or Vangl2 siRNA duplexes (assuming a volume of ~1.6 ml per testis, i.e., 160  pmol siRNA duplexes). Based on results of pilot experiments, a total of 2 transfections were performed to maximize Vangl2 knockdown. The first transfection was done on day 1 and the second transfection on day 5, and rats were euthanized on day 8 by CO_2_ asphyxiation using slow (20–30%/min) displacement of chamber air from a compressed CO_2_ tank. Testes were then removed and processed for immunohistochemistry (IHC) (*n* = 3 rats) wherein testes were fixed in Bouin’s fixative for 24  h, and processed for paraffin embedding as described^43^. Some testes were either snap frozen in liquid nitrogen to obtain frozen cross-sections for IF to visualize F-actin using FITC-phalloidin as described^31^, or used for seminiferous tubule isolation for confocal microscopy. Seminiferous tubules were also isolated from testes as described^44^ for selected experiments for lysate preparations.

### Imaging and 3D reconstruction of isolated seminiferous tubules

**Fig. 1:**
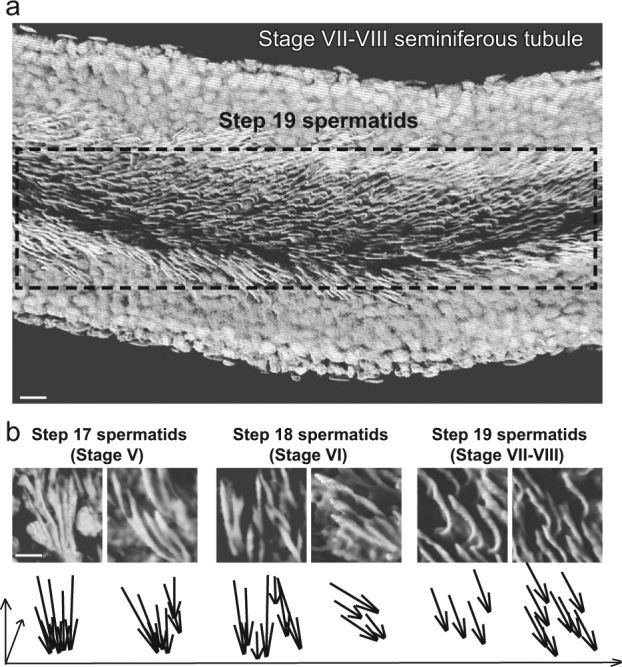
Illustration of spermatid PCP in the seminiferous tubule of adult rat testes by confocal microscopy. The presence of PCP in the testis is supported by the alignment of a field of polarized elongated spermatids within the plane of the seminiferous epithelium in a stage VII-VIII tubule in adult rat testes. **a** A stage VII–VIII seminiferous tubule isolated by using transillumination stereomicroscopy was imaged and 3D reconstructed by confocal microscopy. A white pseudo-color was applied to the DAPI stained cell nuclei in this stage VII–VIII tubule to render optimal contrast to illustrate the PCP alignment of polarized step 19 spermatids (with their heads pointing to the basement membrane) within the plane of the Sertoli cell epithelium. The proximal and distal ends of step 19 spermatid heads (annotated in the dashed box) were found to point unidirectionally to the basement membrane and the tubule lumen, respectively, across the plane of the seminiferous epithelium, exhibiting a PCP phenotype. Scale bar=20 µm. **b** Magnified images of spermatid heads from stage V, VI, and VII–VIII tubules illustrate the alignment of step 17, 18, and 19 spermatid heads, respectively, also supporting spermatid PCP with their heads all pointing toward the basement membrane (annotated by arrowheads in lower panel) either as isolated cells in step 18 and 19 spermatids vs. clusters of step 17 spermatids. Arrows in the lower panel are used to mark the orientation of spermatid heads. Scale bar=6 µm, which applies to other micrographs in the panel

**Fig. 2:**
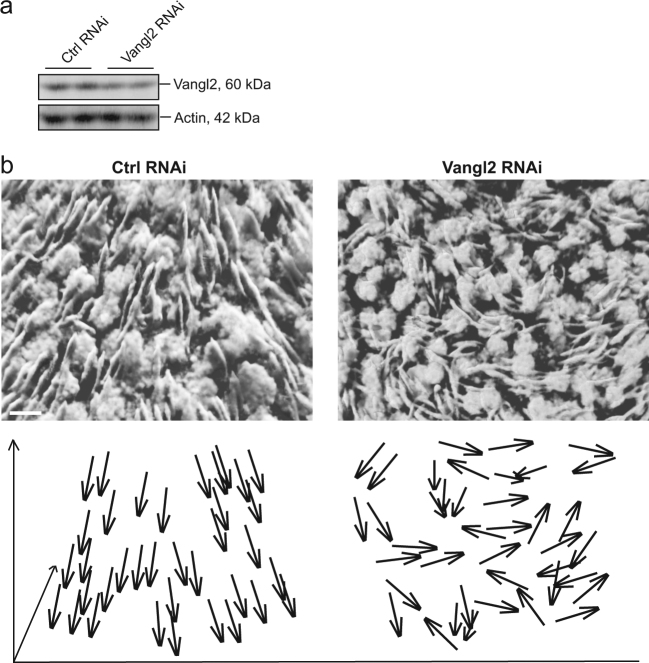
Elongated spermatid PCP in the seminiferous epithelium is perturbed following Vangl2 knockdown in tubules by RNAi. **a** Vangl2 KD in the seminiferous tubules was confirmed by immunoblotting as noted herein. Testes were transfected with either negative non-targeting controls siRNA duplexes (Ctrl) or Vangl2 specific siRNA duplexes at 100 nM (assuming a testis volume of ~1.6-ml) on day 1 and day 5 using PolyPlus in vivo-jetPEI reagent as described in Materials and Methods, and rats were sacrificed on day 8. Testes were removed and processed for immunoblotting using protein lysates or for confocal microscopy shown in (**b**). **b** Stage VII-VIII seminiferous tubules isolated from testes  by transillumination stereomicroscopy and  transfected with either negative non-targeting control or Vangl2 siRNA duplexes were imaged and 3D reconstructed as described in Materials and Methods. A white pseudo-color was applied to the DAPI staining of the cell nuclei to render optimal contrast. Arrows in the lower panel are used to mark the orientation of selected clusters of  spermatid heads, illustrating the PCP phenotype of polarized spermatid heads (annotated by arrowheads in lower panel, pointing to the basement membrane) was grossly perturbed following Vangl2 silencing. Scale bar=10 µm, which applies to the other micrograph

Testes isolated from normal adult rats or rats subjected to Vangl2 RNAi were decapsulated. Seminiferous tubules were collected in a 120-mm culture dish containing fresh F12/DMEM. Staged seminiferous tubules were selected and isolated by transillumination stereomicroscopy at ×10 magnification as earlier described^45^. Isolated seminiferous tubules were collected in 1.7-ml microcentrifuge tubes and fixed in 4% PFA (paraformaldehyde)/PBS (wt/vol) for 1 h. After 3 washes with PBS, seminiferous tubules were permeablized with 0.1% Triton X-100 (vol/vol) and 0.1% Tween 20 (vol/vol) in PBS for 15 min. Thereafter, seminiferous tubules were washed in PBS for three times and were incubated in anti-α-tubulin antibody at 4 °C overnight. Another three washes in PBS, followed by secondary antibody incubation for 1 h, tubules were stained with 4′, 6-diamidino-2-phenylindole (DAPI) and mounted in ProLong® Gold Antifade Mountant (Thermo Fisher Scientific) in MatTek Corp 35-mm glass bottom dishes. Confocal microscopy was performed at the Rockefeller University Bio-Imaging Resource Center. Images were obtained using an inverted Zeiss LSM 880 NLO laser scanning confocal and multiphoton microscope (Carl Zeiss MicroImaging, Thornwood, NY) equipped with the Zeiss ZEN software package. Optical sections of 20–100 µm of the seminiferous tubule were collected at 0.83-µm intervals along the *z*-axis to obtain image series (i.e., Z stack). Autoquant X deconvolution software (Media cybernetics) was used for deconvoluting confocal images. The deconvolved images were then volume rendered and shadow projected 3D in Imaris (Bitplane). Snapshots of the reconstructed seminiferous tubules were taken in Imaris with different magnifications. For images presented in Figs. 1 and 2, the deconvolved images were volume rendered and shadow projected in 3D in Imaris (Bitplane) using only the DAPI channel to show the arrangement of the spermatid heads. White pseudo color was applied to the DAPI channel to render optimal image contrast. For other images presented, deconvovled images of the Z-stack series of the seminiferous tubules were reconstructed in Imaris with the blue channel for DAPI, green channel for α-tubulin and red channel for the Cy3-labeled siRNA duplexes.

### Preparation of Vangl2 mutants and their overexpression in Sertoli cells

Cloning of the Vangl2 full-length cDNA was performed as reported earlier^31^. The plasmid pCI-neo/Vangl2 containing the full-length Vangl2 cDNA served as the template to obtain the deletion mutants using primer pairs shown in Table S1. For instance, deletion of PDZ-binding domain corresponding to the last four amino acid residues of Vangl2 (ETSV, i.e., Glu-Thr-Ser-Val) was obtained by PCR using primer 1 and 2 (Table S1). Deletion of the entire cytoplasmic domain (CD)  of Vangl2 was generated using primer 1 and 3 (Table S1). A start and a stop codon was inserted at the 5′- and the 3′-end, respectively (Table S1). Each mutant had the corresponding *Mlu*l and *Not*l cloning sites at the 5′- and 3′-end, respectively, to allow its cloning into the pCI-neo mammalian expression vector (Promega). The two plasmids were designated pCI-neo/Vangl2∆ETSV and pCI-neo/Vangl2∆CD, respectively. Primary rat Sertoli cells were transfected with the plasmid DNA using K2 Transfection Reagent (Biontex, Munich, Germany) for 14 h on day 2. Thereafter, cells were rinsed with F12/DMEM twice, and cultured in F12/DMEM supplemented with growth factors for an additional 36 h. Cells were terminated on day 4 to obtain cells lysates for immunoblottings, or fixed and processed for dual-labeled immunofluorescence analysis (IF). For IF, plasmid DNA was labeled with Cy3 using a Label IT® Tracker Intracellular Nucleic Acid Localization Kit (Mirus) to illustrate successful transfection

### Treatment of Sertoli cells with PKCζ pseudo-substrate inhibitor (PSI)

PKCζ pseudo-substrate inhibitor (PSI) (Mr 1718), a known specific peptide inhibitor of PKCζ^46,47^ was obtained from Santa Cruz (Dallas, TX). It was dissolved in sterile PBS to obtain a 500 µM stock solution. PKCζ PSI was incubated with Sertoli cell cultures for 24 h at a final concentration of 50 µM. This concentration was established based on pilot experiments and was within the effective dose range of earlier reports^46,47^.

### Microtubule (MT) spin-down assay

Microtubule (MT) spin-down assay to assess the level of polymerized MTs in Sertoli cells was performed as described^43,48^. In short, primary Sertoli cells were seeded on 6-well plates at 0.4×10^6^ cells/cm^2^. For silencing experiments, on day 2 and 4, cells were transfected with non-targeting Ctrl siRNA duplexes vs. Vangl2, MARK2, or Vangl2+MARK2 siRNA duplexes. On day 5, cells were harvested in pre-warmed lysis and MT stabilization buffer (100 mM PIPES, 5 mM MgCl_2_, 1 mM EGTA, 0.1% NP-40, 0.1% Triton X-100, 0.1% Tween 20, 0.1% β-mercaptoethanol, 30% glycerol, pH 6.9). Cells were then homogenized using a 22-gauge syringe needle in 1.7-ml microfuge tubes. In this assay, Taxol (also known as Paclitaxel, a MT stabilizing agent^49^) at 30 µM and CaCl_2_ at 4 mM (known to induce MT depolymerization^50^) were added onto cell lysates of Sertoli cells treated with negative non-targeting control siRNA duplexes (Ctrl RNAi) to serve as the corresponding positive and negative controls. Thereafter, cells were centrifuged at 100,000 × *g* for 30 min at 35 °C to separate free tubulin monomers (supernatant) from polymerized tubulins (pellet; i.e., MTs). Supernatant was collected, 20 μl supernatant of each sample were analyzed by immunoblotting for β-tubulin. Pellet was re-suspended in 250 μl of 4 mM CaCl_2_ in MilliQ water, and 20 μl of each sample were used for immunoblotting. For overexpression experiments, on day2, cells were transfected with pCI-neo empty vector vs. either pCI-neo+PKCζ PSI, pCI-neo/Vangl2 or pCI-neo/Vangl2+ PKCζ PSI. On day 4, cells were collected and processed, as described above.

### Immunofluorescence (IF) and dual-labeled immunofluorescence analysis

Cross-sections (~7-µm thick) of frozen adult rat testes obtained in a cryostat at −22 °C and Sertoli cells cultured on cover-glass were fixed with either ice-cold methanol (for Vangl2, PKCζ and α-tubulin cell staining) or 4% PFA (wt/vol) (for all others) in PBS, and permeabilized with 0.1% Triton X-100 (vol/vol) in PBS. Testis sections and Sertoli cells were then blocked with 1% BSA (wt/vol) at room temperature, and were incubated overnight at 4 °C with primary antibodies using dilutions ranging from 1:50 to 1:200 (Table S2), to be followed by an hour incubation with corresponding secondary antibodies (1:250 for cross-sections of testes and 1:100 for cells) at room temperature. For Vangl2 immunofluorescence staining, whole testes were fixed in 3.7% paraformaldehyde (wt/vol) in PBS, cryo-preserved in gradient sucrose (20–30% in PBS) and subsequently embedded in paraffin. Thereafter, cross-sections were obtained, de-paraffinized, and rehydrated. Antigen retrieval was performed in citric acid buffer (10 mM sodium citrate, pH 6.0 at 22 °C) for ~10 min in a microwave oven. Thereafter, cross-sections were processed for fluorescence staining as described above. For F-actin staining, FITC-conjugated phalloidin (Invitrogen-Thermo Fisher Scientific) was used. Nuclei were visualized with 4′, 6-diamidino-2-phenylindole (DAPI). Images were obtained using an Olympus BX61 fluorescence microscope with a built-in Olympus DP-71 digital camera and images were acquired using the Olympus MicroSuite Five software package (Version 1224). Images were analyzed using Adobe Photoshop for image overlay.

### Immunoblot analysis

Immunoblotting was performed as described^51^. In brief, ~15–50 µg protein of Sertoli cell lysates or ~30 µg protein lysates of seminiferous tubule lysates were used for SDS-PAGE. Proteins were then electroblotted onto nitrocellulose membranes for analysis using corresponding antibodies listed in Table S2.

### Statistical analysis

Each data point was expressed as a mean ± SD of at least three independent experiments or *n* = 3 rats. Statistical significance was evaluated with Student’s *t-*test for paired comparisons. One-way ANOVA followed by Tukey’s test was used for group comparison.

## Results

### Vangl2 regulates spermatid planar cell polarity (PCP) in the testis

In mammalian testes, Sertoli cells are associated with germ cells at different developmental stages along the seminiferous tubules^4,5^. These associations progress in highly organized cycles throughout spermatogenesis known as the cycle of seminiferous epithelium. One cycle is divided into different stages based on the morphologically distinctive cellular associations^52^, with 14 stages in the rat^4,5^, and 12 in the mouse^36,53^. For instance, the release of sperm takes place at stage VIII of the cycle, coinciding with the appearance of step 8 spermatids and the preleptotene spermatocytes in the seminiferous epithelium. PCP refers to the orderly alignment of a field of polarized cells within the plane of an epithelium such as those found in wing cell hair in *Drosophila* and cell hair of the inner ear in mammals^15,23,32^. Using confocal microscopy, developing spermatids in particular step 17–19 spermatids in stage V–VIII tubules were found to display PCP in the reconstructed 3D image such as the one shown in Fig. 1a, b, analogous to cell hair in cochlea. For instance, as noted in the schematic drawings shown in the lower panel of Fig. 1b wherein each spermatid in the seminiferous epithelium of a control tubule was annotated by an arrow, a field of polarized spermatids, resembling cell hair in cochlea, was found to have their heads pointing toward the basement membrane (see upper panel in Fig. 1b). Knockdown of Vangl2, a PCP protein earlier shown to be expressed by Sertoli cells and also germ cells in adult rat testes^31^, by ~60% (Fig. 2a) in seminiferous tubules was found to induce gross disruption of PCP in step 19 spermatids in stage VII and early stage VIII tubules as shown in Fig. 2b. The loss of PCP penotype was also depicted in the schematic drawing in the lower panel of Fig. 2b, wherein some randomly selected spermatids, including many whose PCP was grossly affected, were shown with arrows. Successful transfection was monitored by visualizing Cy3-labeled (red fluorescence) Vangl2-specific siRNA duplexes in the testis (Figure S1A). These findings thus support the notion that Vangl2 is involved in spermatid PCP during spermatogenesis in adult rat testes.

### Vangl2 co-localizes with MTs and PKCζ in the seminiferous epithelium of adult rat testes

**Fig. 3:**
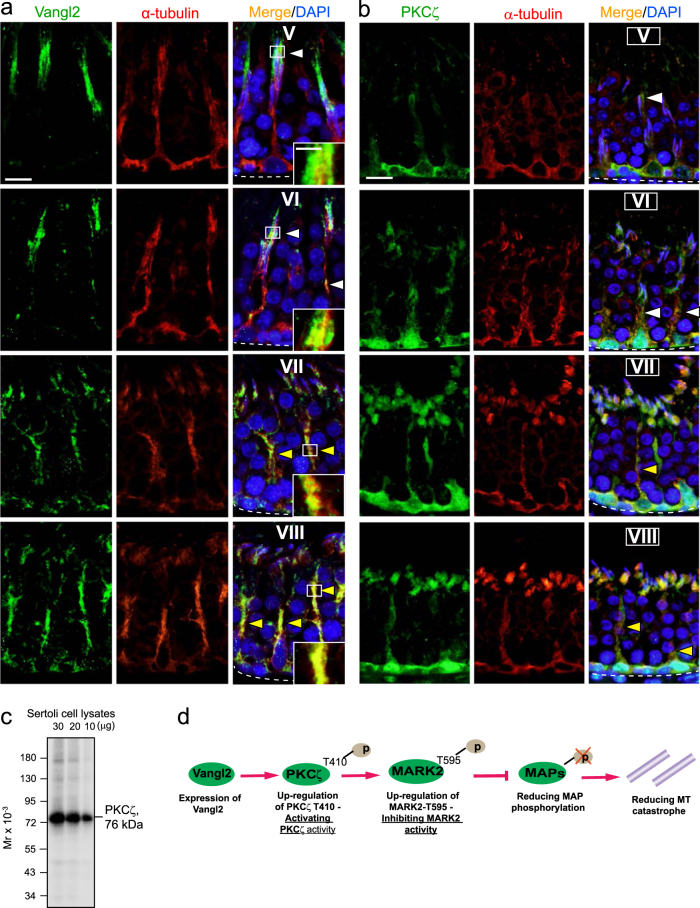
Co-localization of Vangl2 with microtubule (MT) in the seminiferous epithelium during the epithelial cycle of spermatogenesis. **a** Cross-sections of adult rat testes, illustrating tubules from stage V-VIII of the epithelial cycle, were stained for Vangl2 (green fluorescence) and α-tubulin (red fluorescence, which together with ß-tubulin are the building blocks of MTs). Vangl2 was shown only to partially co-localize with α-tubulin (white arrowheads) at the sites of step 17 and 18-spermatid and Sertoli cell interface in stage V and VI seminiferous tubules, respectively. However, these two proteins were found  to become better co-localized along the stalk-like structures of the MT network (yellow arrowheads) in stage VII and VIII tubules, illustrating co-localization of Vangl2 and MTs was stage-specific. Scale bar=20 µm, which applies to all other micrographs in this panel; inserts showed magnified images from the respective boxed areas, scale bar=10 µm, which applies to other inserts. **b** Results of a study based on dual-labeled immunofluorescence microsocopy, illustrating partial co-localization of PKCζ (green fluorescence) and α-tubulin (i.e., MTs; red fluorescence) (see white arrowheads) in stage V–VI tubules, but better co-localization of these two proteins in stage VII–VIII tubules (see yellow arrowheads). Scale bar, 20 µm, which applies to other micrographs. **c** Results of immunoblotting to illustrate the specificity of the anti-PKCζ antibody (see Table S2) using declining protein lysates of Sertoli cells, indicating the apparent Mr of PKCζ at 79 kDa. **d** A schematic drawing illustrating the downstream signaling proteins utilizing by Vangl2 to modulate PCP in other mammalian tissues and/or cells including PKCζ^37,38^, and the involvement of this signaling pathway was investigated in subsequent experiments

MTs are known to organize as track-like strucutres in the testis, similar to those in other tissues^54,55^, by aligning perpendicular to the basement membrane across the seminiferous epithelium to support the transport of spermatids and organelles (e.g., residual bodies, phagosomes) during the epithelial cycle as reported^35,43^. Herein, Vangl2 (green fluorescence) was shown to partially co-localize with α-tubulin (red fluorescence) (note: α and ß-tubulins are the building blocks of MTs) (Fig. 3a). Furthermore, α-tubulin also co-localized with signaling protein PKCζ(Fig. 3b), using a specific anti-PKCζ antibody (Fig. 3c; Table S2). Vangl2 was found to co-localize better with MTs in stages VII–VIII tubules (Fig. 3a) as noted in the enlarged images in Fig. 3a. These findings illustrate that Vangl2 may be associated with MTs and may also be structurally related to the signaling protein PKCζ. These data are important since they support the notion that Vangl2 and PKCζ may be working in concert to regulate MT dynamics. This observation coupled with earlier reports regarding the involvement of PKCζ and MARK2 in PCP protein dishevelled signaling function^37,38^ thus prompted us to hypothesize that Vangl2 activated PKCζ through an up-regulation of p-PKCζ-Thr410, to be followed by an increase in p-MARK2-Thr595 expression (Fig. 3d). This up-regulation of p-MARK2-Thr595 inactivated MARK2, making it incapable of phosphorylating MAPs (microtubule associated proteins, such as MAP1a), which in turn reducing MT catastrophe and stabilizing MTs^56,57^ (Fig. 3d).

### Vangl2 knockdown in the testis perturbs MT- and F-actin-based organization, down-regulating the expression of phosphorylated form of signaling proteins PKCζ and MARK2

**Fig. 4:**
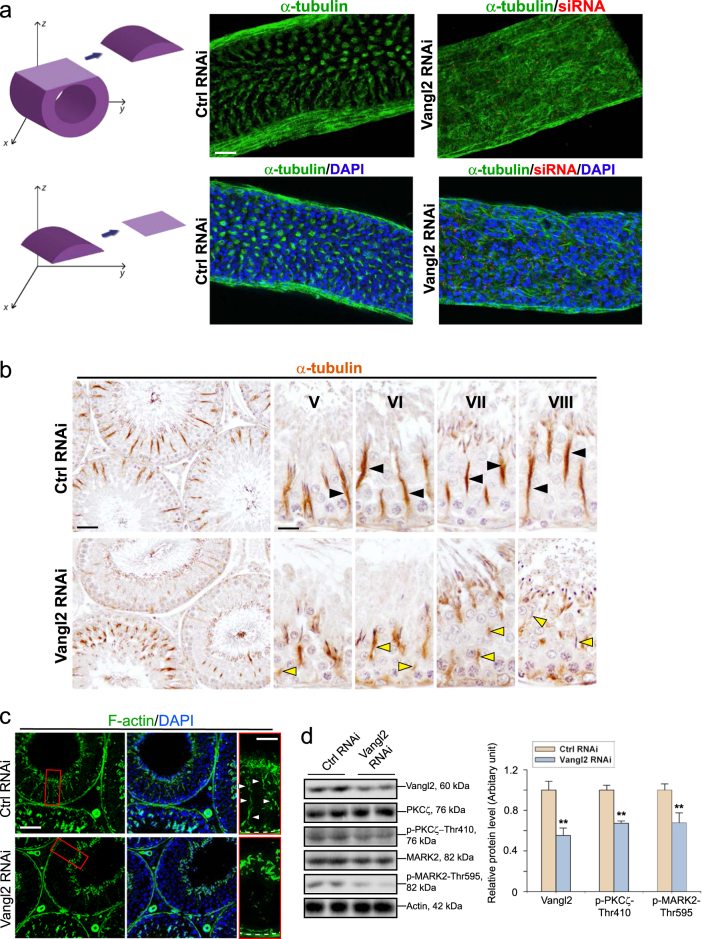
Vangl2 knockdown in the testis in vivo grossly disrupts MT organization in the seminiferous epithelium. **a** 3D reconstructed image by confocal microscopy of a section of seminiferous tubule (right, top panel) (see illustrations on the left panel, depicting views from the *z*-axis) transfected with either non-targeting control siRNA duplexes (Ctrl RNAi) vs. Cy3 labeled-Vangl2 siRNA duplexes (red fluorescence; Vangl2 RNAi), illustrating the organization of MT (visualized as green fluorescence using an anti-α-tubulin antibody). A representative scanned image selected from the z-series of a seminiferous tubule was shown in the right, lower panel wherein cell nuclei were stained with DAPI. It was noted that MT organization was grossly disrupted following Vangl2 knockdown in vivo. Scale bar, 40 µm, which applies to all other micrographs. **b** A study by immunohistochemistry, illustrating gross disruption of MT (brownish precipitate of α-tubulin immunostaining) organization in the seminiferous epithelium in all stages of the epithelial cycle and representatives findings from stages V-VIII tubules are shown herein. For instance, in control testes, MT-conferred tracks laid across the seminiferous epithelium and aligned perpendicular to the basement membrane. However following Vangl2 knockdown, these MT-based tracks were truncated, thereby failing to support spermatid transport so that step 19 spermatids were also seen embedded deep inside the epithelium (see yellow arrowheads). These findings are consistent with data shown in **a**, illustrating the stalk-like structures conferred by MTs were grossly disrupted following Vangl2 knockdown in vivo. Scale bar, 50 µm, for the low (and corresponding) magnified cross-sections of testes; and 20 µm, which applies to higher (and corresponding) magnified micrographs of staged tubules. **c** A parallel study was performed by immunofluorescence analysis to assess disruptive effects of Vangl2 knockdown on the organization of F-actin in the seminiferous epithelium. The F-actin-conferred tracks notably found in stage VIII tubules in control testes (Ctrl RNAi) were absent in similarly staged tubules following Vangl2 knockdown (Vangl2 RNAi), as noted in the enlarged images boxed in red. Scale bar, 70 µm for the first panel, and 30 µm for the last panel, which apply to corresponding images in the same panel. **d** A study by immunoblot analysis using lysates of seminiferous tubules isolated from testes of Vangl2 RNAi vs. Ctrl RNAi group, illustrating a knockdown of Vangl2 also induced a down-regulation of p-PKCζ Thr410 (but not total PKCζ) and p-MARK2-Thr595 (but not total MARK2) in tubules (left panel; see also composite data in Figure S2A for total PKCζ and MARK2). This down-regulation of p-MARK2-Thr595 rendered MARK2 incapable of phosphorylating MAPs, reducing MT catastrophe to make MT to become less dynamics. ß-actin served as a protein loading control. Histogram on the right panel is a summary of the IB findings shown on the left panel (see also Figure S2A). Each bar is a mean ± SD of *n* = 3 independent experiments. **P<0.01 by Student’s *t*-test

3D views on changes in the organization of MTs in seminiferous tubules (Fig. 4a, upper panel) and also changes in MT organization relative to the PCP alignment of elongated spermatids (Fig. 4a, lower panel) following Vangl2 knockdown vs. control tubules. These changes were further confirmed using cross-sections of testes from Vangl2 knockdown testes vs. control testes wherein the organization of MTs was assessed by immunohistochemistry (IHC) using a specific α-tubulin antibody (Table S2). It was noted that the prominent MT-based tracks across the seminiferous epithelium that laid perpendicular to the basement membrane found in control testes (Fig. 4b) were virtually broken into short fragments and some of them no longer aligned perpendicular to the basement membrane in Vangl2 knockdown testes (Fig. 4b). These disruptive changes in MT organization induced by Vangl2 knockdown thus impeded spermatid transport so that elongated spermatids were found to be embedded deep inside the epithelium in tubules wherein spermiation had taken place (see yellow arrowhead that annotated trapped elongated spermatids in Fig. 4b). Similarly, F-actin organization was also considerably disrupted following Vangl2 knockdown in the testis in vivo, in which the track-like structures conferred by F-actin microfilaments, typically found in late stage VIII tubules were virtually absent (Fig. 4c; see also the enlarged images). Interestingly, the activated/phosphorylated form of PKCζ (but not total PKCζ) designated p-PKCζ-Thr410 was down-regulated (Fig. 4d and Figure S2A). p-PKCζ was earlier shown to negatively regulate MARK2 activity by phosphorylating MARK2 on Thr595^58^. It is noted that MARK2 is known to phosphorylate MT-associated proteins (e.g., MAP1a) to induce MT catastrophe, causing MT destabilization and its subsequent breakdown^59^. Thus, a down-regulation of the phosphorylated form of MARK2, namely p-MARK-Thr595 (but not total MARK2) (Fig. 4d, Figure S2A), as the result of a reduction in p-PKCζ-Thr410 would promote MARK2 activity, leading to MT catastrophe in response to changes in the epithelial cycle to support spermatogenesis. As such, changes in the expression of p-PKCζ-Thr410 and p-MARK2-Thr595 following Vangl2 knockdown led to defects in spermatogenesis as noted in Fig. 4b, consistent with results of an earlier report^31^. For instance, the loss of MT-based tracks that disrupted spermatid transport led to  retention of elongated spermatids in stage VIII tubules when spermiation had taken place (Fig. 4b). Furthermore, the findings that a down-regulation on the expression of p-PKCζ-Thr410 and p-MARK2-Thr595 following Vangl2 knockdown in the testis (Fig. 4d) was also confirmed using Sertoli cells cultured in vitro following Vangl2 knockdown (Figure S1B) and was noted in Figure S1C.

### Vangl2 knockdown impairs MT organization through considerable reduction in MT polymerization in Sertoli cells

**Fig. 5:**
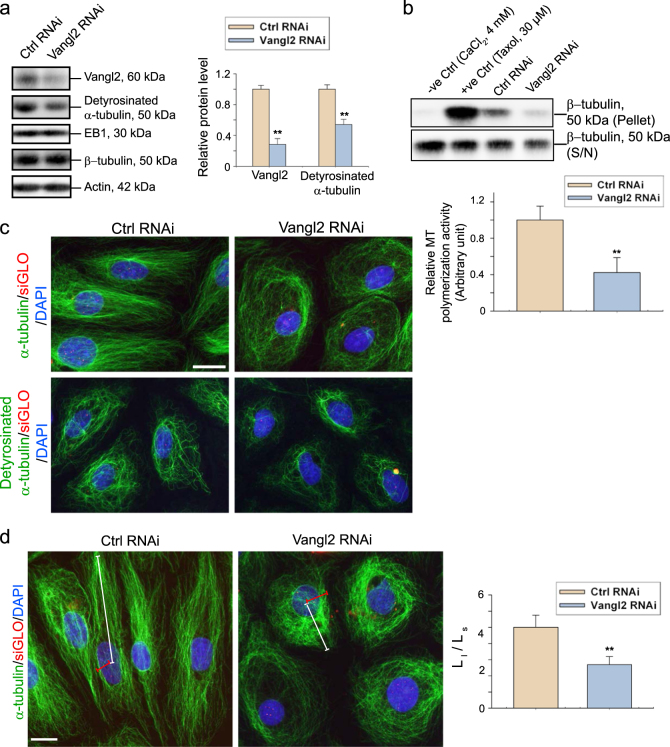
Knockdown of Vangl2 in vitro perturbs MT organization in Sertoli cells through changes in MT polymerization activity. **a** Knockdown of Vangl2 by RNAi using specific Vangl2 siRNA duplexes (Vangl2 RNAi) vs. the non-targeting control siRNA duplexes (Ctrl RNAi) was assessed by immunoblotting wherein the expression of Vangl2 was reduced by ~70%, but also reducing the expression of detyrosinated α-tubulin by ~40% (note: detyrosinated α-tubulin is known to confer MT stabilization, making MT less dynamic). However, the expression of EB1, a +TIP protein known to stabilize MTs, did not alter, with ß-tubulin and ß-actin served as the protein loading control. Some data shown in the left panel were summarized in the bar graph and shown on the right. Each bar is a mean ± SD of *n* = 3 independent experiments. **P<0.01 by Student’s *t*-test. **b** MT spin-down assay was performed using lysates of Sertoli cells transfected with either non-targeting negative control or Vangl2 siRNA duplexes to assess the relative level of polymerized MTs in Sertoli cells following Vangl2 knockdown. Sertoli cells treated with CaCl_2_ (4 mM) and Taxol (30 µM) served as the corresponding negative and positive control, in which CaCl_2_ is known to promote MT depolymerization whereas Taxol is a MT stabilizing reagent. A considerable decline in the β-tubulin (note: α- and ß-tubulin serve as the building blocks of MT) level following Vangl2 knockdown in the pellet illustrates that Vangl2 is involved in promoting MT polymerization and/or MT stabilization. The histogram below summarizes results of immunoblot analysis such as those shown above with each bar represents a mean ± SD of *n* = 3 independent experiments using different batches of Sertoli cells. **P<0.01 by Student’s *t*-test compared to control. **c** IF analysis was performed using Sertoli cells transfected with either non-targeting control or Vangl2 siRNA duplexes wherein considerable changes in MT organization were detected in Vangl2 silenced cells by α-tubulin staining. For instance, MTs no longer stretched across the entire Sertoli cell cytosol but retracted from cell periphery, moving closer to encircle the cell nucleus. A considerable down-regulation of the fluorescent intensity of detyrosinated a-tubulin following Vangl2 knockdown was observed, consistent with findings shown in (**a**). Successful transfection was indicated by DY-547-siGLO (red fluorescence; Dharmacon-GE Healthcare) which was used for co-transfection with siRNA duplexes. Scale bar, 25 µm, which applies to other micrographs. **d** Changes in MT organization in Sertoli cells following Vangl2 knockdown was quantified by comparing the longest distance of MT visualized by α-tubulin staining from the cell periphery to the nucleus (L_l_) with the shortest one (L_s_) in 70 randomly selected cells from an experiment. Scale bar=20 µm, which applies to other micrographs. A statistically significant difference in the ratio from the Vangl2 knockdown cells vs. control cells was noted in the histogram shown on the right panel. Each bar is a mean ± SD of *n* = 3 experiments using different batches of Sertoli cells. **P<0.01 by Student’s *t*-test

Successful transfection in cultured Sertoli cells was monitored by visualizing Cy3-labeled Vangl2-specific siRNA duplexes (Figure S1B). Following Vangl2 knockdown in Sertoli cells by ~70%, there was no detectable changes in the expression of EB1 (end binding protein 1, a plus (+) end tracking protein, +TIP, known to induce MT stabilization at the fast growing (+) end^60^) (Fig. 5a). However, a consistent and considerable down-regulation of detyrosinated-α-tubulin was noted (Fig. 5a). Detyrosinated α-tubulin refers to the removal of C-terminal Tyr by exposing Glu at the newly formed C-terminus of α-tubulin, known to induce MT stabilization by rendering MT less dynamics^61–63^. Thus, MTs in Sertoli cells following Vangl2 knockdown that led to a down-regulation of detyrosinated-α-tubulin were less stable. It was also observed that knockdown of Vangl2 in cultured Sertoli cells led to a decrease in the steady-state level of p-PKCζ-Thr410 (but not total PKCζ) and p-MARK2-Thr595 (but not total MARK2) (Figure S1C). This was consistent with in vivo data shown in Fig. 4d and Figure S2A. Based on results of a biochemical experiment by monitoring the level of polymerized MTs in Vangl2 knockdown vs. control Sertoli cells, it was noted that Vangl2 knockdown led to a considerable reduction in MT polymerization (Fig. 5b—see both upper and lower panel). In this assay, Sertoli cell lysates used for MT polymerization assessment also incubated with either CaCl_2_ (4 mM)^64^ or Taxol (30 µM)^65,66^ at the specified concentration as reported to inhibit and promote MT polymerization, by serving as the corresponding negative and positive control, respectively (Fig. 5b, upper panel). ß-tubulins in the supernatant (S/N) were non-polymerized/free tubulins and the pellet represented polymerized MTs. When the organization of α-tubulin and detyrosinated α-tubulin across the Sertoli cell cytosol was examined in Vangl2 knockdown vs. control cells, a considerably diminished detyrosinated α-tubulin was noted (Fig. 5c), consistent with immunoblotting data shown in Fig. 5a. Furthermore, representative findings from three independent experiments support the notion that the organization of MTs and of detyrosinated α-tubulin was grossly disrupted following Vangl2 knockdown in which the spindle-shaped MTs that stretched across the Sertoli cell cytosol found in controls were encircling the cell nuclei, assuming a round-shaped organization (Fig. 5c). These changes in MT organization were semi-quantitatively analyzed and shown in Fig. 5d by measuring the ratio between the length from the longest end of MTs to the cell nucleus (L_l_) and the length from the shortest end of MTs to the cell nucleus (L_s_) from 50 randomly selected cells in each experiment with *n* = 3 independent experiments, indicating significant changes in the organization of MTs following Vangl2 knockdown.

### Vangl2 exerts its regulatory effects on signaling proteins PKCζ and MARK2 through the C-terminal cytoplasmic domain, independent of the PDZ-binding domain

**Fig. 6:**
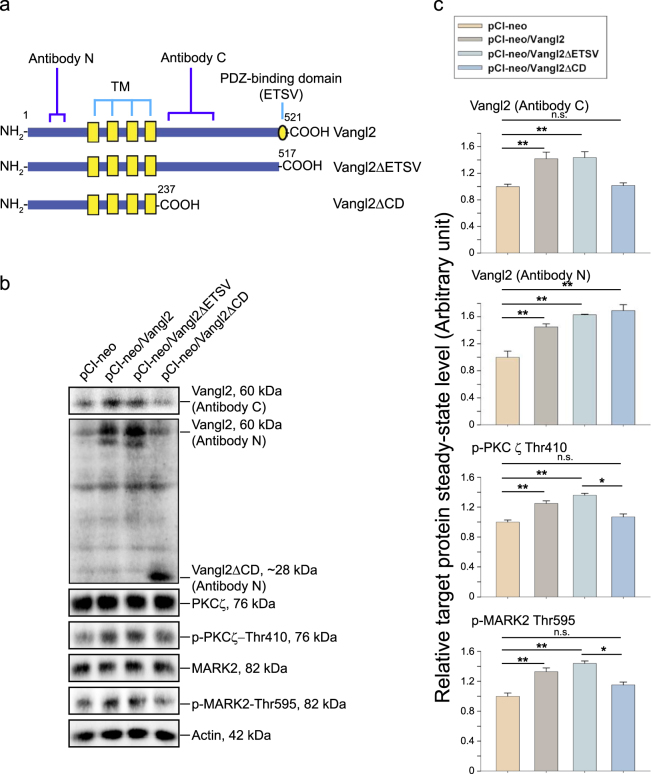
A study to assess the functional domains at the C-terminal cytoplasmic region of Vangl2 to modulate signaling function of PKCζ and MARK2. **a** A schematic drawing illustrates the two deletion mutants of Vangl2: Vangl2∆ETSV with the deletion of the PDZ-binding domain of amino acid residues ETSV (i.e., deletion of residues 518 to 521) from the C-terminus as a 517 residues protein, and Vangl2∆CD with the deletion of the entire C-terminal cytoplasmic region of 280 amino acids and the PDZ-binding domain of 4 amino acids (i.e., deletion of 284 residues) as a 237 residues protein vs. the full-length Vangl2 clone of 521 amino acid residues. Two anti-Vangl2 antibodies with one targeting the N-terminal cytoplasmic domain (Antibody N, from Santa Cruz, see Table S2) and the other targeting the C-terminal cytoplasmic region (Antibody C, from Sigma-Aldrich, see Table S2) were also indicated. These mutants were prepared by PCR as described in Materials and Methods using the corresponding primer pairs shown in Table S1. TM, transmembrane domain. **b** Overexpression of Vangl2 and its two corresponding mutants was confirmed by immunoblotting shown in the first two panels using the corresponding anti-N and anti-C terminal region antibody (see Table S2). More important, overexpression of Vangl2 and Vangl2∆ETSV induced an up-regulation of p-PKCζ-Thr410, which is a hallmark of an activation of atypical PKC (aPKC, note: aPKC is comprised of PKCζ and PKCλ) activity. It also induced an up-regulation of p-MARK2-Thr595, which is known to inactivate MARK2^58,75^. However, overexpression of Vangl2∆CD did not induce aforementioned phenotype including either a surge in expression of either p-PKCζ-Thr410 or p-MARK-Thr595, illustrating the effects of Vangl2 on aPKC and MARK2 activity was probably mediated through the C-terminal cytoplasmic region of Vangl2, independent of the PDZ-binding domain. **c** These bar graphs summarize results of immunoblotting data shown in **b**. Each bar is a mean ± SD of *n* = 3 independent experiments using different batches of Sertoli cells. See also composite data for total PKCζ and total MARK2 following immunoblotting and shown in Figure S2B. *P<0.05; **P < 0.01; using one-way ANOVA followed by Tukey’s test; n.s., not significantly different

We next assessed the functional domain in Vangl2 that is involved in down-regulating p-PKCζ-Thr410 and p-MARK2-Thr595 after Vangl2 knockdown in Sertoli cells. A functional Vangl2 molecule is composed of a C-terminal PDZ-binding domain of Glu-Thr-Ser-Val (ETSV) and a long stretch of sequence of the cytoplasmic domain (CD) of 280 amino acid residues (Fig. 6a). In short, we prepared two Vangl2 deletion mutants: (i) Vangl2∆ETSV by deleting the C-terminal PDZ-binding domain of ETSV, and (ii) Vangl2∆CD by deleting 284 amino acid residues containing the entire cytoplasmic domain (280 amino acids) and the PDZ-binding domain (4 amino acids). These two deletion mutants and the full-length Vangl2 cDNA were overexpressed in Sertoli cells. Lysates were obtained and immunoblot analysis was performed using an antibody against an epitope near the C-terminal region within the cytoplasmic domain (designated Antibody C, from Sigma-Aldrich) vs. an antibody against an epitope near the N-terminal region (designated Antibody N, from Santa Cruz) (see Table S2). Immunoblot analysis using these two antibodies confirmed the expression of the corresponding mutant proteins in Sertoli cells (Fig. 6b). It is of interest to note that only overexpression of full-length Vangl2 and deletion mutant Vangl2∆ETSV were capable of inducing an up-regulation of p-PKCζ-Thr410 (but not total PKCζ) and p-MARK2-Thr595 (but not MARK2) (Fig. 6b, c and Figure S2B). These findings coupled with the data shown in Fig. 4d and Figure S2A based on analysis following Vangl2 knockdown thus support the notion that the stretch of sequence within the cytoplasmic domain of 280 amino acids is responsible for regulating PKCζ and MARK2 function in Sertoli cells. We next investigated the involvement of PKCζ and MARK2 signaling proteins in Vangl2 function.

### Vangl2 regulates MT dynamics to modulate spermatid PCP function through PKCζ and MARK2 signaling proteins in the testis

**Fig. 7:**
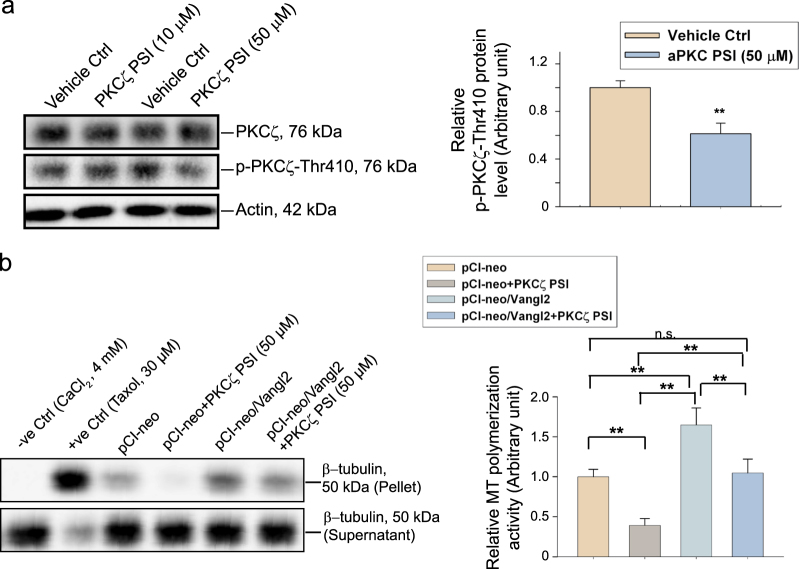
A study by utilizing specific inhibitor and overexpression of Vangl2 full-length cDNA to illustrate that Vangl2 regulates Sertoli cell MT organization through signaling protein PKCζ downstream. **a** Sertoli cells  (0.04  x 10^6^ cells/cm^2^) cultured alone for 3 days were incubated with PKCζ pseudo-substrate inhibitor (PSI), a specific inhibitor for PKCζ at 10 or 50 µM for 24 h. Cells were then terminated to obtain lysates for IB using corresponding antibodies (Table S2). At 50 µM, PSI was found to down-regulate p-PKCζ-Thr410 (the activated form of PKCζ) expression by ~40% (see bar graph on right panel), which was the concentration used for subsequent experiments. Each bar is a mean ± SD of *n* = 3 independent experiments. **P<0.01 by Student’s *t*-test. **b** Results of a representative biochemical MT polymerization assay to illustrate that treatment of Sertoli cells with PKCζ-specific PSI (50 µM) or overexpression of Vangl2 in Sertoli cells blocked or up-regulated the ability of these cells to induce MT polymerization as noted in the cell pellet, respectively. The presence of PKCζ-PSI was effective to negate the stimulatory effect of Vangl2 overexpression on MT polymerization activity (left panel). CaCl_2_ (4 mM) and Taxol (30 µM) included in the Sertoli cell extracts served as the corresponding negative and positive controls in the MT polymerization assay. Free tubulins or short stretches MTs were found in the supernatant (S/N) whereas polymerized MTs were detected in the pellet. These findings were summarized in the bar graph shown on the right panel with each bar representing a mean ± SD of *n* = 3 independent experiment using different batches of Sertoli cell cultures. Each experiment had at least triplicate cultures. **P<0.01

**Fig. 8:**
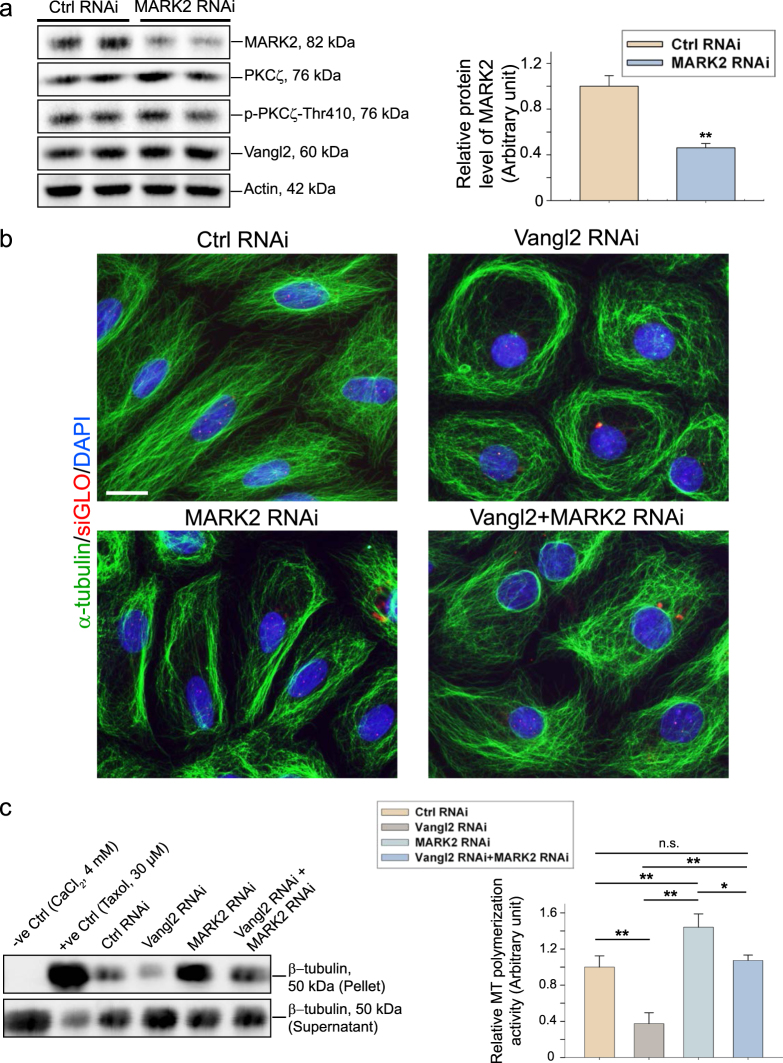
A study by utilizing Vangl2 and MARK2 RNAi versus overexpression of Vangl2 to illustrate that Vangl2 regulates Sertoli cell MT organization through signaling protein MARK2 downstream. **a** Immunoblotting using lysates of Sertoli cells following transfection with MARK2 specific siRNA duplexes for MARK2 knockdown vs. the non-targeting negative control siRNA duplexes. The bar graph in the right panel summarizes immunoblotting data shown on the left panel to confirm MARK2 expression was knocked down by at least ~60% without altering the expression of neither PKCζ, PKCζ-Thr410, nor Vangl2 (ß-actin served as a protein loading control). Each bar is a mean ± SD of *n* = 3 independent experiments. **P<0.01 by Student’s *t*-test. **b** Vangl2 knockdown by RNAi induced disorganization of MT network in Sertoli cells, consistent with data shown in Fig. 5c, d. MARK2 knockdown, however, did not cause considerable changes in the phenotypes of Sertoli cells except that more MTs were noted that stretched across the Sertoli cell cytosol at or near  the cell cortical zone. However, a double knockdown of Vangl2 and MARK2 in Sertoli cells was shown to block the disruptive effects caused by Vangl2 knockdown alone, at least in part, since these double knockdown Sertoli cells no longer assumed a rounded configuration and MTs could stretched across the Sertoli cell cytosol. Successful transfection was indicated by DY-547-siGLO (red fluorescence; Dharmacon-GE) which was used for co-transfection with siRNA duplexes. Scale bar=20 µm, which applies to other micrographs. **c** Results of a representative biochemical assay to assess the ability of Sertoli cell lysates to induce MT polymerization. Following Vangl2 RNAi, a considerable reduction in MT polymerization was noted; however, MARK2 RNAi considerably induced the ability of Sertoli cells to induce MT polymerization. Moreover, a double knockdown of Vangl2 and MARK2 was found to rescue the disruptive effects of Vangl2 RNAi-induced reduction in MT polymerization. Bar graph on the right panel is a summary of the data shown on the left panel. Each bar is a mean ± SD of *n* = 3 independent experiments using different batches of Sertoli cells, and each experiment had triplicate cultures

PSI (pseudosubstrate inhibitor of PKC), earlier shown to block PKCζ activation^67^, was selected in our experiments at 50 µM, within the range of 50–100 µM known to selectively block PKCζ activation and not the other isoforms (e.g., PKCλ)^46,47^. Indeed, PSI at 50 µM blocked PKCζ activation since the steady-state level of p-PKCζ-Thr410 was considerably down-regulated following treatment of Sertoli cells with PSI (Fig. 7a). However at 10 µM, PSI had no effect in blocking PKCζ activation (Fig. 7a), consistent with an earlier report^46^. In a study based on the use of  the MT polymerization assay, overexpression of Vangl2 in Sertoli cells was found to induce MT polymerization, whereas the presence of PSI considerably inhibited the Vanlg2-induced MT polymerization. Moreover, PKCζ PSI alone also blocked Sertoli cell MT polymerization activity effectively (Fig. 7b). Collectively, these findings support the notion that PKCζ is a downstream signaling molecule of Vangl2-mediated MT regulation. We next examined the role of MARK2 on Vangl2-mediated function on MT organization based on the likely signaling pathway of Vangl2 shown in Fig. 3d. To probe the involvement of MARK2 in Vangl2 signaling, we first silenced MARK2 in Sertoli cells by RNAi (Fig. 8a). The knockdown of MARK2 in Sertoli cells by ~60%  did not induce any changes in the expression of PKCζ, p-PKCζ-Thr410 nor Vangl2 illustrating MARK2 knockdown did not induce any off-target effects (Fig. 8a). However, MARK2 knockdown was found to restore MT organization induced by Vangl2 knockdown when both Vangl2 and MARK2 were silenced by transfecting Sertoli cells with the corresponding siRNA duplexes vs. non-targeting negative control siRNA duplexes (Fig. 8b). On the other hand, MARK2 knockdown in Sertoli cells by RNAi induced MT polymerization capability, and double knockdown of MARK2 and Vangl2 was found to rescue Sertoli cells from the Vangl2 knockdown-induced down-regulation of MT polymerization activity (Fig. 8c). Taking collectively, these findings support the hypothetical signaling pathway depicted in Fig. 3d that Vangl2 indeed exerts its regulatory effects on MT organization through signaling proteins PKCζ (Fig. 7) and MARK2 (Fig. 8) downstream.

## Discussion

Using confocal imaging and 3D reconstruction of the seminiferous tubules, we have confirmed the presence of PCP in the testis, regarding the alignment of polarized spermatids on the plane of seminiferous epithelium^28^. This type of polarity represented by the alignment of step 18-19 spermatids, which display uniformed orientation of the curved spermatid heads at the proximal (i.e., spermatid nuclei) end of the cells across the seminiferous epithelium, resembling PCP in the inner ear. Interestingly, although the apico-basal polarity is detectable in elongating spermatids as early as at steps 8-11 during spermiogenesis when apical ES becomes the only anchoring device during spermiogenesis, the PCP-like phenotype is fully discernable in step 19 elongated spermatids in VII–VIII tubules. These observations also suggest that the PCP-like phenotype requires the support of other regulatory proteins besides the classical apico-basal polarity protein complexes, namely the Par^10^, the Scribble^11^, and the Crumbs-based^12^ polarity protein complexes recently identified in the testis. In this report, we provide compelling evidence that the PCP protein Vangl2 is an important contributing protein to confer spermatid PCP. For instance, a knockdown of Vangl2 by ~60–70% by RNAi was found to grossly perturb spermatid PCP when examined by 3D reconstructed images from confocal microscopy. Work is also in progress to identify the roles of other PCP proteins (e.g., Prickle 1, Dishevelled 3) which are known to work in concert with Vangl2 in other species (e.g.*, Drosophila*) in conferring and regulating spermatid PCP during spermiogenesis. While we focus the current report on the role of MT-based cytoskeleton on Vangl2-mediated spermatid PCP, our earlier report has demonstrated that Vangl2 exerts its effects on actin-based cytoskeleton^31^. Herein, we also confirmed in the same experiment, a knockdown of Vangl2 indeed perturbed the organization of actin-based cytoskeleton, such as the F-actin-conferred tracks were considerably diminished in Vangl2 knockdown testes. This disruptive effect has been previously shown to be mediated by actin regulatory proteins Arp3 and Eps8^31^. Thus, it is likely that both cytoskeletons are involved in Vangl2-mediated spermatid PCP. Findings reported herein have supported the concept that the Vangl2-mediated effects on MTs were regulated, at least in part, by two downstream signaling molecules PKCζ and MARK2. Moreover, their activities rely on the 280 amino acid residues in the C-terminal region of Vangl2. However, it remains to be investigated if the signaling proteins PKCζ and MARK2 are also involved in Vangl2-mediated F-actin re-organization.

As noted herein, a loss of Vangl2 function by RNAi was found to reduce MT stability as illustrated by a declining detyrosinated α-tubulin expression, and a reduced MT polymerization activity in Sertoli cells. This observation is consistent with an earlier study reporting that a loss of Vangl2 function reduced the acetylated MT (a form of stable MT) in *Xenopus* oocytes^38^. Also, Vangl2 was found to modulate aPKC activity in Sertoli cells. For instance, knockdown of Vangl2 by RNAi impeded the expression of p-PKCζ -Thr410 considerably. We speculate that the effects of Vangl2 on PKCζ activity may be an indirect cellular event. It was reported that dishevelled (Dvl), another PCP protein, interacted with PKCζ directly, capable of inducing PKCζ activity through the phosphorylation of PKCζ at Thr410 ^37^. Dvl was earlier shown to facilitate axon formation of hippocampal neurons in studies in vitro by stabilizing and activating PKCζ, which in turn inhibited MARK2 activity, thereby promoting MT stability^37^. Vangl2, a small transmembrane protein is known to recruit cytoplasmic Dvl to specific domains near the cell membrane for PCP signal transduction^68^. Translocation of Dvl from cell cytosol towards the plasma membrane is a prerequisite for PCP signal activation in both *Drosophila* and vertebrates^69–71^. Also, Vangl2 interacts with Dvl via its C-terminal region independent of the PDZ-binding domain^72^. Herein, it was shown that overexpression of either Vangl2 full-length cDNA (wild type) or the PDZ-binding domain-deletion mutant was capable of inducing PKCζ activation but not overexpression of Vangl2 mutant which lacked the entire C-terminal cytoplasmic region. These findings thus illustrate that the C-terminal cytoplasmic region excluding the PDZ-binding domain of Vangl2 may play a determining role in regulating PKCζ activity. Thus, it is likely that Vangl2 modulates PKCζ activity, at least in part, through its interaction with Dvl. It is possible that when overexpressed in Sertoli cells, the ectopic expressed Vangl2 recruits more Dvl to its membrane location, activating PCP signaling and inducing PKCζ activity. The activated PKCζ thus phosphorylates MARK2 at Thr595, inhibiting its activity on MAP proteins, thereby stabilizing MT organization. The possible involvement of Dvl in Vangl2 signaling deserves additional investigations.

In summary, Vangl2 is a putative PCP regulatory protein in the testis that confers spermatid PCP during spermiogenesis through its effects on PKCζ and MAPK2 downstream, by modulating the organization of Sertoli cell MTs. Work is now in progress to determine the role of Prickle, the binding partner of Vanlg2, and the involvement of Frizzle (a PCP integral membrane protein) and Dvl (the binding partner of Frizzle)^73,74^ in spermatogenesis and their functional relationship with Vangl2.

## Electronic supplementary material

Suplementary Material
