# Supplementary material for: Vangl2 regulates spermatid planar cell polarity through microtubule (MT)-based cytoskeleton in the rat testis
Source: Cell Death Dis. 2018 Mar 1;9(3):340. doi: 10.1038/s41419-018-0339-x (PMC5832773; doi:10.1038/s41419-018-0339-x)
Supplement: Supplementary file 1 — Suplementary Material [file 41419_2018_339_MOESM1_ESM.pdf]

Supplementary Material

Chen H et al.

Vangl2 regulates spermatid planar cell polarity through microtubule (MT)-based cytoskeleton in the rat testis

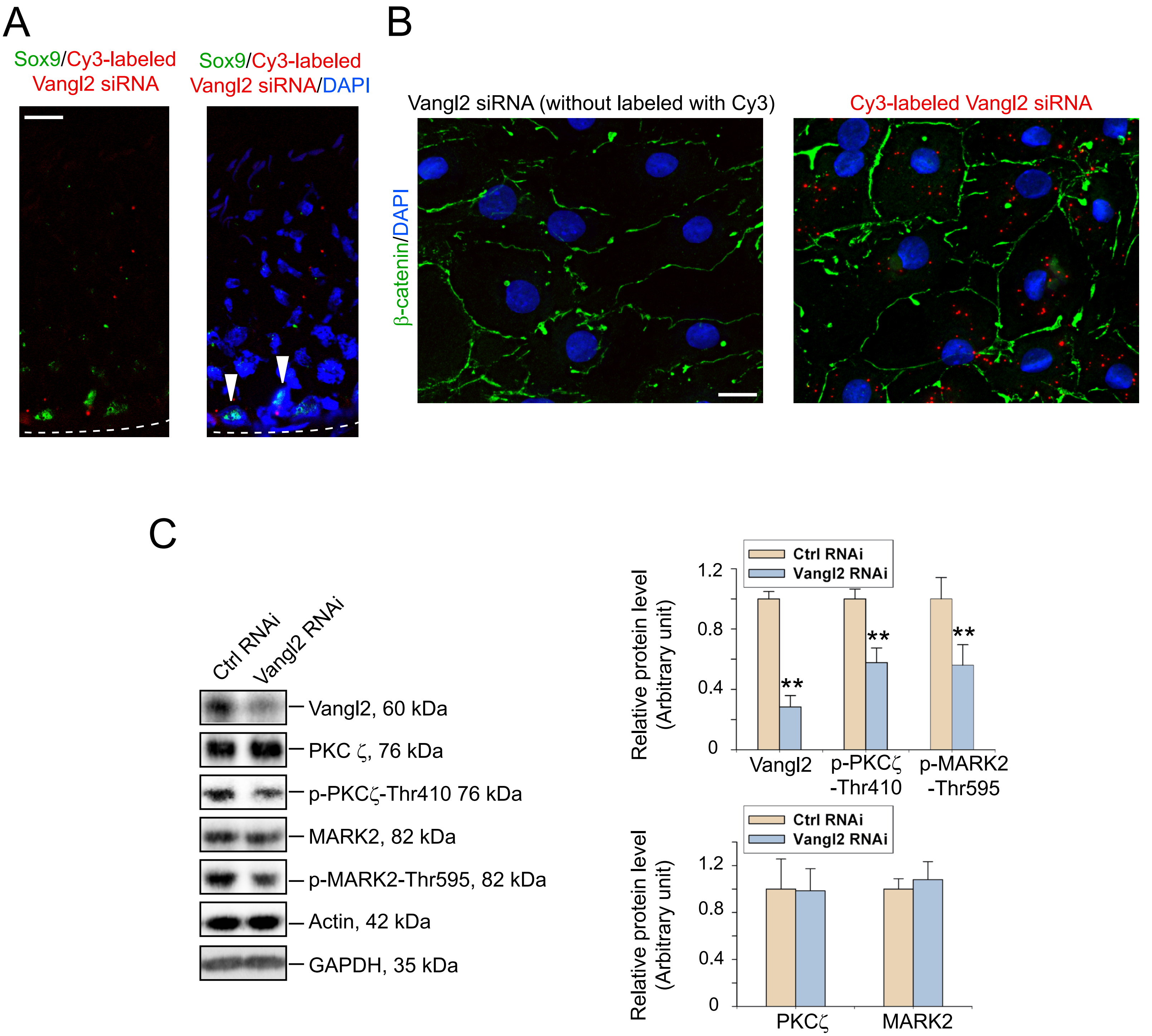

Figure S1. A study to assess successful transfection of testes in vivo and Sertoli cells in vitro with Vangl2 siRNA duplexes to assess changes in the expression of selected signaling proteins following Vangl2 knockdown. A). Immunofluorescence analysis (IF) of frozen cross-sections of testes following transfection with Cy3-labeled Vangl2-specific siRNA duplexes (red fluorescence) to knockdown down Vangl2, wherein Sertoli cells were visualized by Sox9 (green fluorescence, a Sertoli cell-specific transcription factor in the testis). Note that siRNA duplexes (red fluorescence) were predominantly accumulated near Sertoli cell nuclei (white arrowheads). Cell nuclei were stained with DAPI. The relative location of the basement membrane in the seminiferous epithelium was annotated with a white dashed line. These findings thus support data shown in Figure 4, confirming successful transfection of the testis with Vangl2 siRNA duplexes. Scale bar, 20 µm, which applies to the other micrograph. B). IF of cultured Sertoli cells following transfection with Vangl2-specific siRNA duplexes without labeling vs. Cy3-labeled Vangl2-specific siRNA duplexes (red fluorescence). β-catenin (green fluorescence, a Sertoli cell BTB adaptor protein known to be localized at the cell-cell interface, forming an adhesion protein complex with N-cadherin) was stained to visualize Sertoli cell cortical region at the cell-cell interface. Note that siRNA duplexes (red fluorescence) were predominantly accumulated in Sertoli cell cytosol following successful transfection. Sertoli cell nuclei were stained with DAPI. Scale bar, 40 µm, which applies to the other micrograph. C). A study by immunoblot analysis using lysates of cultured Sertoli cells transfected with Vangl2-specific (Vangl2 RNAi) vs. non-targeting negative control siRNA duplexes (Ctrl RNAi), illustrating a knockdown of Vangl2 by ~70% induced a down-regulation of p-PKCζ-Thr410 (but not total PKCζ) and p-MARK2-Thr595 (but not total MARK2). This finding was consistent with in vivo data shown in Figure 4D. Both ß-actin and GAPDH served as protein loading controls. Histograms on the right panel are a summary of the IB data shown on the left panel. Each bar is a mean±SD of n = 3 independent experiments. **, $P<0.01$ by Student's t-test.

A

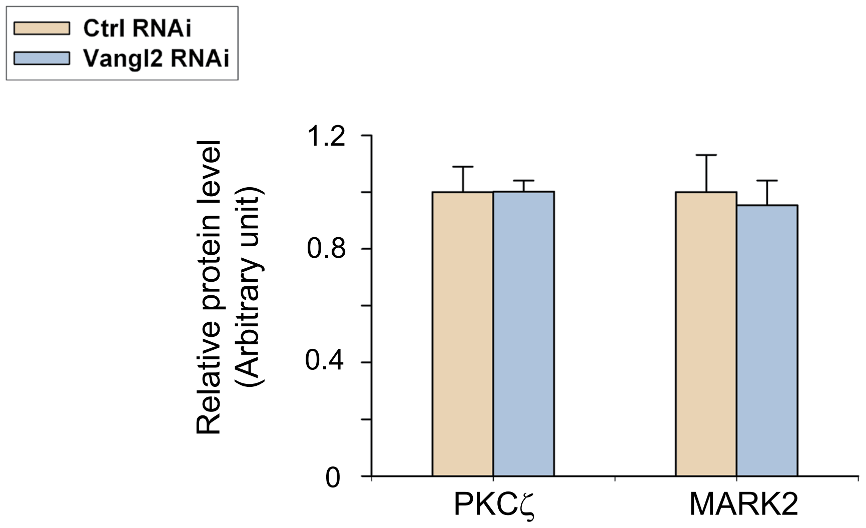

B

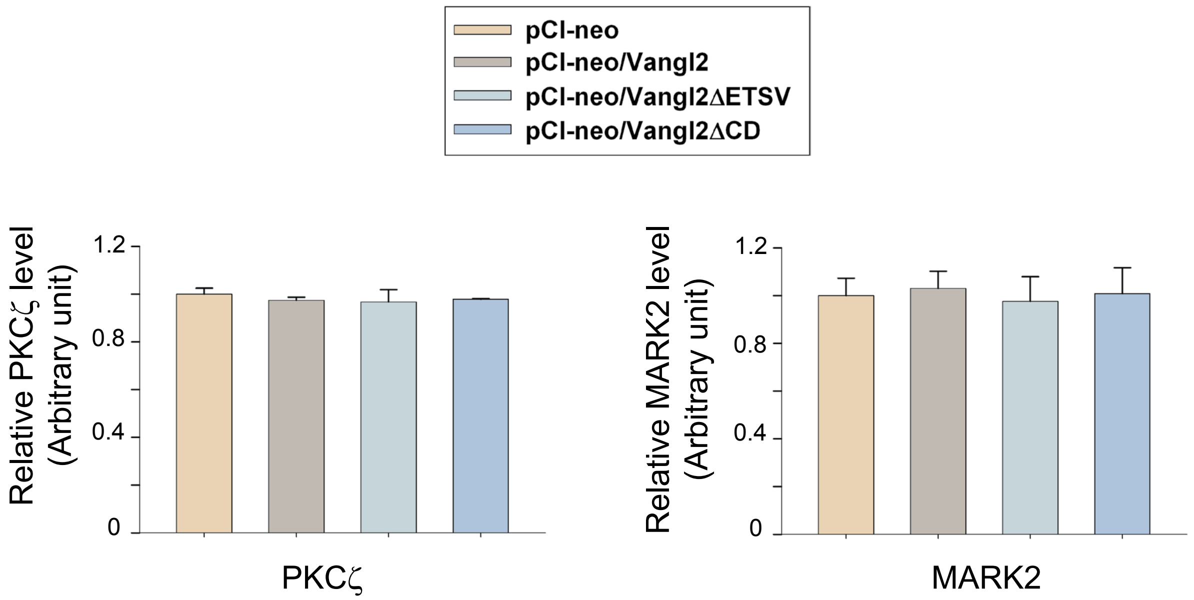

Figure S2. Immunoblot analysis to assess changes in the steady-state levels of signaling proteins following knockdown of Vangl2 in the testis in vivo (A) or overexpression of Vangl2 vs. its deletion mutants in Sertoli cells in vitro (B). A). Histogram summarizing results noted in Figure 4D on the relative protein levels of total PKCζ and total MARK2 in the testis following Vangl2 knockdown (i.e., Vangl2 RNAi) vs. Ctrl RNAi using data as noted in the left panel. Each bar is a mean±SD of *n* = 3 independent experiments. B). Histogram summarizing results noted in Figure 6B on the relative protein levels of total PKCζ and total MARK2 in Sertoli cells following overexpression of empty vector (Ctrl) vs. Vangl2 WT and two deletion mutants in cells cultured in vitro. Each bar is a mean±SD of *n* = 3 independent experiments.

Table S1. Primers used for cloning experiments in this report

No.	Primer Name	Primer orientation	Primer sequence (5'-3')
1	Vangl2-S	Sense	CA*ACGCGT*ATGGACACCGAGTCCCA
2	Vangl2ΔETSV-AS	Anti-Sense	AT*GCGGCCGC*TCACGACTGCAGCCGCATGA
3	Vangl2ΔCD-AS	Anti-sense	AT*GCGGCCGC*TCACAGCAGAACTACGGCCA

The restriction sites are *Mlu*I (italicized sequence in primer 1) at the 5'-end and *Not*I (italicized sequence in primer 2 and 3) at the 3'-end. Both the start (ATG) and stop (TGA, i.e., TCA in antisense primer) codons were underlined. Nucleotide sequences of constructs were confirmed by direct nucleotide sequencing at Genewiz.

Table S2. Antibodies used for various experiments in this report.

Antibody (RRID)	Host species	Vendor	Catalog no.	Working dilution IB	Working dilution IF
Actin (AB_630836)	Goat	Santa Cruz Biotechnology	sc-1616	1:300	
α-tubulin (AB_2241226)	Mouse	Abcam	ab7291	1:1000	1:300
β-tubulin (AB_2210370)	Rabbit	Abcam	ab6046	1:1000	
β-catenin (AB_2533039)	Rabbit	Thermo Fisher Scientific	13-8400		1:100
GAPDH (AB_2107448)	Mouse	Abcam	ab8245	1:1000	
p-MARK2Thr595 (AB_11037367)	Rabbit	Novus Biologicals	NBP1-78028	1:250	
p-PKCζ-Thr410* (AB_2168217)	Rabbit	Cell Signaling Technology	9378	1:1000	
PKCζ (AB_2300359)	Rabbit	Santa Cruz Biotechnology	sc-216	1:1000	1:200
Vangl2 (AB_10548021)	Rabbit	Sigma-Aldrich	HPA027043	1:200	
Vangl2 (AB_2213082)	Goat	Santa Cruz Biotechnology	sc-46561	1:500	1:50 (cells)
Vangl2 (AB_2272693)	Sheep	R&D Systems	AF4815		1:100 (testes)
Sox9 (AB_2255399)	Mouse	Santa Cruz Biotechnology	sc-166505		1:100
Rabbit IgG-HRP(AB_634837)	Bovine	Santa Cruz Biotechnology	sc-2370	1:3000	
Mouse IgG-HRP (AB_634824)	Bovine	Santa Cruz Biotechnology	sc-2371	1:3000	
Rabbit IgG-Alexa Fluor 555 (AB_2535850)	Goat	Thermo Fisher Scientific	A-21429		1:100 (cells)<br>1:250 (testes)
Mouse IgG-Alexa Fluor 488 (AB_2534088)	Goat	Thermo Fisher Scientific	A-11029		1:100 (cells)<br>1:250 (testes)
Mouse IgG-Alexa Fluor 555 (AB_2535844)	Goat	Thermo Fisher Scientific	A-21422		1:100 (cells)<br>1:250 (testes)
Sheep IgG-Alexa Fluor 488 (AB_141362)	Donkey	Thermo Fisher Scientific	A-11015		1:100 (cells)<br>1:250 (testes)

*This antibody also cross-reacted with p-PKCλ–Thr403 besides p-PKCζ-Thr410 as indicated by the manufacturer.
